# Exploring maternal and neonatal outcomes in women with Type-1 Diabetes: A study from Pakistan

**DOI:** 10.12669/pjms.40.7.9199

**Published:** 2024-08

**Authors:** Musarrat Riaz, Saima Askari, Raheela Naseem

**Affiliations:** 1 Musarrat Riaz, FCPS (Med), FCPS (Endo) Associate Professor, Department of Medicine, Consultant Endocrinologist, Baqai Institute of Diabetology and Endocrinology, Baqai Medical University, Karachi, Pakistan; 2 Saima Askari, FCPS (Med), FCPS (Endo) Assistant Professor, Department of Medicine, Consultant Endocrinologist, Baqai Institute of Diabetology and Endocrinology, Baqai Medical University, Karachi, Pakistan; 3 Raheela Naseem Diabetes Educator, DDE, Baqai Institute of Diabetology and Endocrinology, Baqai Medical University, Karachi, Pakistan

**Keywords:** Pregnancy outcomes, T1DM, Materno-fetal complications, Preconceptional education, Unplanned pregnancies

## Abstract

**Background & Objective:**

Pregnancy in women diagnosed with Type-1 diabetes mellitus poses a higher risk of experiencing complications related to the health of the fetus, the mother, and the newborn, along with potential obstetric issues. The objective of this study was to examine the maternal and fetal outcomes, as well as complications faced by pregnant women with type-1 diabetes, and to identify potential preventable factors.

**Methods:**

This retrospective cohort study, conducted at Baqai Institute of Diabetology and Endocrinology (BIDE), Baqai Medical University, Karachi, Pakistan (January 2022 - January 2023), focused on registered pregnancies of women with Type-1 diabetes. A predesigned questionnaire recorded demographic information, diabetes and obstetric history, clinical details, treatment specifics, maternal, perinatal, and neonatal outcomes.

**Results:**

This study included 100 women with pre-existing Type-1 diabetes (mean age: 15.11 ± 5.64 years at diabetes diagnosis). Of these, 72% reported unplanned pregnancies, with a mean HbA1C at conception 8.29%. Median gestational age at delivery was 32.15 ± 10.82 weeks. Delivery outcomes included 40% normal vaginal deliveries and 60% C-sections (9% emergency, 51% elective). Stillbirths occurred in 14 cases, while 16 women experienced one miscarriage, seven had two, and 10 had three miscarriages. Glycemic targets (fasting) were achieved in 55 women, and post-meal targets only in 29, whereas, neonatal complications included hypoglycemia in 13 and low birth weight in 12 neonates.

**Conclusion:**

The high frequency of unplanned pregnancies and cesarean sections along with poor management of pre-pregnancy care and poor glycemic control results in compromised maternal and perinatal outcomes in this high-risk group.

## INTRODUCTION

The prevalence of Type-1 diabetes in pregnant women has been increasing, ranging from 1.56 to 4.09 per 1000 pregnancies in recent years.^1^ This rise in cases poses significant challenges in managing pregnancies with Type-1 diabetes, as it is associated with numerous complications for both the mother and the fetus. Reports indicate a high prevalence of comorbidities and complications in young women with Type-1 diabetes.^2^ They have 2-3 fold increase in mortality rates.^3^ Neonatal complications like macrosomia, respiratory distress syndrome, Neonatal Intensive Care Unit (NICU) admissions, intrauterine growth restriction, congenital anomalies, premature birth, neonatal hypoglycemia and metabolic disorders. have been observed in pregnancies with Type-1 mothers.^4^ Also, there is a strong correlation between elevated HbA1C and the risk of congenital malformations in neonates of pregnant women with Type-1 diabetes. Consequently, high blood glucose concentrations are considered a significant teratogen, contributing to these adverse outcomes.^5^

A study conducted in Taiwan highlighted strong associations between Type-1 diabetes and various adverse outcomes. These outcomes included pregnancy-related hypertension, preeclampsia, eclampsia, cesarean delivery, stillbirth, preterm birth, adult respiratory distress syndrome, pulmonary edema, sepsis, chorioamnionitis, puerperal cerebrovascular disorders, acute renal failure, and shock.^6^ Furthermore, data from another study indicated that 73% of pregnancies with Type-1 diabetes had unfavorable outcomes and interestingly, all of these pregnancies were unplanned. Additionally, 47% of women with Type-1 diabetes had pre-existing diabetes-related complications.^7^

A recent systematic review has indicated that adverse outcomes in pregnant women with Type-1 diabetes can be mitigated through preconception care, planned conception, optimal glycemic control, folic acid supplementation, and management of other diabetic vascular complications. Therefore, offering preconception counseling and treating medical comorbidities while optimizing HbA1c levels can enhance perinatal mortality and morbidity outcomes.^8^ Limited research in South Asia, particularly Pakistan, on pregnancy outcomes in Type-1 diabetes is attributed to inadequate follow-ups and insufficient coordination among healthcare providers. Low Middle-Income Countries (LMIC) like Pakistan faces multiple challenges in providing care to women with T1 diabetes. Therefore, the objective of this study was to investigate maternal and neonatal outcomes as well as complications of pregnancy in women with Type-1 diabetes presenting at a tertiary care diabetes center in Pakistan. The secondary aim was to identify potential preventable factors which can modify the outcome of these pregnancies.

## METHODS

This Retrospective cohort study was conducted at Baqai Institute of Diabetology and Endocrinology (BIDE), Baqai Medical University, Karachi, Pakistan over a period of one year from January 2022 to January 2023. Data was retrieved from the records available on Hospital Management System (HMS) and utilized a data set comprising of registered pregnancies of women with Type-1 diabetes who met the predefined inclusion criteria. Women diagnosed with T1DM and who became pregnant at least once after diagnosis of Type-1 diabetes, visiting the outpatient department of BIDE were included. Women with gestational diabetes or those who were diagnosed with Type-1 diabetes during pregnancy and Type-2 diabetic pregnant females were excluded from the study. Convenient sampling was used.

### Ethical Approval:

Following the approval of the Institutional Review Board (IRB). (IRB letter NO 006/06/22/0158)

A predesigned questionnaire was developed for data recording, such as demographic details, diabetes and obstetric history, clinical examination findings, treatment details, and maternal, perinatal, and neonatal outcomes.

### Statistical Analysis:

Descriptive statistics were employed to analyze the data by using SPSS version 22. Mean and standard deviation were calculated for continuous variables. Frequencies were computed for categorical variables.

### Operational Definitions:

*1. Perinatal mortality* refers to the loss of a fetus after 24 weeks of gestation or a baby weighing less than or equal to 500 grams at birth

*2.Postnatal death:* Deaths occurs within seven days after birth

*3.Neonatal hypoglycaemia:* defines as neonatal hypoglycaemia less than 2.6 mmol/l and severe hypoglycaemia as less than 2.0mmol/l

*4. Abortion*, the expulsion of a fetus from the uterus before its viable around 20th week of gestaion

*5.Low birth weight (LBW):* is defined as a birth weight of less than 2500 g (up to and including 2499 g), as per the World Health Organization (WHO)

*6. Stillbirth:* defined as the death or loss of a baby before or during delivery

*7. Miscarriage:* defined as the loss of a pregnancy before 20 weeks’ gestation.

## RESULTS

One hundred and fifteen women became pregnant during the study period. Out of which complete record of 100 women was available for analyses. The mean age at the time of diagnosis of type 1 diabetes was 15.11+ 5.64 years. Mean duration of diabetes before pregnancy was 10.81±7.56 while age at time of first pregnancy was 21.95± 3.79 years respectively. Mean HbA1C at the time of conception was 8.29%. The data obtained shows that 51% of pregnant women were counselled about glycemic targets before conceiving, however among them unplanned pregnancies were reported in 72 (72%) women. In 1st trimester, 90% of subjects had taken folic acid regularly, (Table-I). The median gestational age at delivery was 32.15±10.82 weeks.

**Table-I:** Demographics and baseline characteristics of pregnant women with type-1 diabetes.

Parameters	Frequency (%) or mean ± SD
Number of women	100
Age at T1DM diagnosis (years)	15.11±5.64
Age at the time of first pregnancy (years)	21.95±3.79
Duration of diabetes before pregnancy (years)	10.81±7.56
***Number of pregnancies***	
1	15
2	24
3	21
4	23
> 4	17
Median gestation age at delivery (weeks)	32.15±10.82
***Pregnancy Status***	
Planned	28 (28%)
	72 (72%)
HbA1c at the time of conception (%)	8.29±1.66
Mean fasting blood glucose during pregnancy (mg/dl)	130.48±62.65
Mean random blood glucose during pregnancy (mg/dl)	215.76±94.62
***Pregnancy-related counseling done prior to pregnancy***	
Yes	70 (70%)
No	30 (30%)
***Preconception advice given***	
Yes	51 (51%)
No	49 (49%)

**Fig.1:**
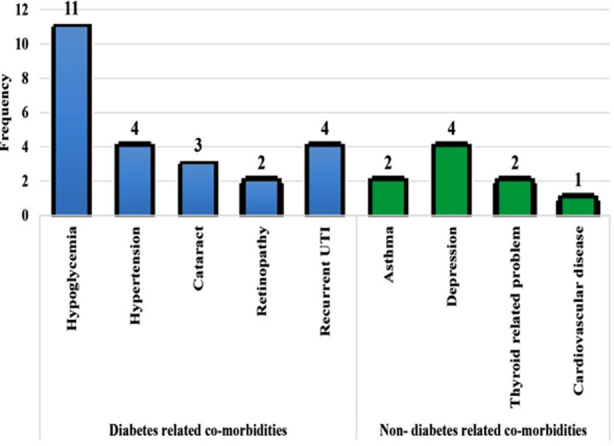
Maternal diabetes and non-diabetes related co-morbidities.

Forty women (40%) underwent normal vaginal delivery whereas 60 women (60 %) had C-section out of which only 9% were emergency cesarian and 51% were elective as shown in Table-II. Most of the women had unplanned pregnancies with poor glycemic control. Keeping this in mind, c-sections were advised to prevent peri-natal complications. Furthermore, maternal diabetes related and unrelated co-morbidities are detailed in Fig.1 which included episodes of minor hypoglycemia in 11%, Hypertension and recurrent UTI in 4% and cataract in 3% women.

**Table-II:** Characteristics observed during the course of pregnancy.

Parameter	Frequency
***Folic acid intake in 1st trimester***	
Yes	90
No	10
***Glycemic targets during pregnancy known***	
Yes	72
No	28
***Contraception discussed***	
Yes	52
No	48
***Antenatal complications***	
No	81
Hypertension	14
UTI	4
Thyroid	1
***Mode of delivery***	
Normal vaginal delivery	40
Caesarean	60
Emergency	9
Optional	51

**Fig.2:**
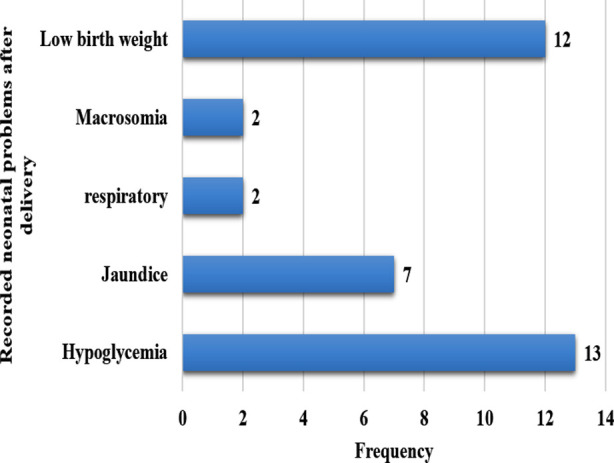
Neonatal problems after delivery.

Regarding pregnancy outcomes, 14 women had stillbirths whereas, 16 women had one miscarriage, seven had two and 10 women had three miscarriages respectively. Glycemic targets (fasting) were achieved in 55 women and post meals in 29 women only, as depicted in Table-III. Regarding neonatal complications, hypoglycemia occurred in 13, and low birth weight in one, jaundice in seven and macrosomia in two neonates as showed in Fig.2.

**Table-III:** Obstetric history of pregnant women with T1DM.

Parameters	Frequency
***No. of babies born alive***	
0	23
1	18
2	23
3	18
More than 3	13
***No. of babies born dead (stillbirth)***	
0	76
1	14
***No. of miscarriages***	
0	62
1	16
2	7
3	10
***No. of abortion***	
0	81
1	9
2	3
***Glycemic targets achieved during pregnancy: Fasting***	
Yes	55
No	33
***Glycemic targets achieved during pregnancy: Post Meals***	
Yes	29
No	58
***Post-natal advice and feeding patterns***	
Breastfeed	44
Bottle feed	15
Both	8

## DISCUSSION

The present study focuses on the maternal, perinatal, and neonatal outcomes of pregnancy in women with pre-existing type 1diabetes. Our data show that (72%) of pregnancies were unplanned, the median gestational week at delivery was 32 weeks, and (60 %) of recruited females underwent caesarian section and among them majority opted for elective caesarian section. Furthermore, the mean HbA1c was 8.29% at the time of conception and stillbirths occurred in 14 cases, while 16 women experienced one miscarriage, seven had two, and 10 women had three miscarriages.

In our study, the mean age at first pregnancy was 21.95 ±3.79 years and the duration of diabetes before pregnancy was10.81±7.56 years. These findings are in contrast to previously published studies that showed the mean age of 29.5 ± 5.42^9^ and 28 years^6^, respectively and the duration of diabetes 14.1 ± 8.05 years.^9^

According to the Confidential Enquiry into Maternal and Child Health (CEMACH), women who have unplanned pregnancies are more likely to experience poor pregnancy outcomes.^10^ Moreover, evidence suggests that prenatal care attendance is linked to lower HbA1c levels in the early stages of pregnancy and a lower frequency of unfavorable outcomes, such as congenital abnormalities, preterm birth, and perinatal mortality.^11^ A majority of participants 3/4th had an unplanned pregnancy in the present study, in agreement to our findings published data showed an almost same frequency of unplanned pregnancy. In contrast, 2017 cohort research by Wotherspoon et al. found that among 747 T1DM women, 39% indicating that their pregnancies were unplanned. A smaller proportion of women with unplanned pregnancies received counseling before pregnancy compared to those who planned their pregnancies. Additionally, babies born to women with unplanned pregnancies were more likely to be smaller than expected for their gestational age.^12^

Folic acid 5mg/day until 12 weeks of pregnancy is recommended by National Institute for Health and care Excellence (NICE)^13^ and in our study, 90 % of women took folic acid in 1st trimester however, National Pregnancy in Diabetes (NPID) audit showed usage in only 41.8%.^14^ Similarly, another study found that only 21.7% of their participants used folic acid at the time of conception.^15^ The strict glycemic control decreases the occurrence and severity of T1DM complications and may improve pregnancy outcomes, especially at the time of conception, according to the diabetes control and complications trial.^16^ However, in our study the mean HbA1c at the time of conception, was 8.29±1.66%, mean fasting and random plasma glucose in mg/dl were 130+62.65 and 215.76±94.62 respectively.^17^ In our study, 3/5th of included women (60 out of 100) delivered via cesarean section (c-section) and among them a majority (51) opted for elective c-section. Similarly, several studies observed that modes of delivery via cesarean section are more prevalent in diabetic mothers.^18^,^19^ Furthermore, the median gestational age was 32.15±10.82 weeks in the present study. Whereas in 2019 American College of Obstetrics and Gynecologists (ACOG) recommended gestational age of 36 to 39 weeks, at which delivery is appropriate for pregnancies complicated by diabetes.^20^

Type-1 diabetes is associated with several complications and other disorders; in our study cohort one/fifth of women had already known comorbidities prior to entering pregnancy, including hypertension, urinary tract infection and thyroid disorder. Various studies have shown that hyperglycemia has influenced and accelerated diabetes induced complications in pregnant women with diabetes especially retinopathy, nephropathy, preeclampsia, hypoglycemia, diabetic ketoacidosis (DKA), and urinary tract infection (UTI), among many others.^21^,^22^ However, in contrast our result reveals that 11% pregnant women had hypoglycemia and 4% women had recurrent UTI and only 2% women developed retinopathy whereas none developed DKA or preeclampsia despite uncontrolled sugars.

Perinatal mortality has been observed to be several folds higher in diabetic mothers as compared to the general population.^23^ In agreement, we found a large number of miscarriages/abortions in approximately half of the studied women and 14 stillbirths in our result. Additionally neonatal problems encountered after delivery observed hypoglycemia, low birth weight, macrosomia, jaundice and respiratory problems in our study in alignment with several other studies that showed the above-mentioned neonatal problems are often seen in people with T1DM, especially with poorer glycemic control.^24^

### Limitations

This was a single-centered study, posing challenges in data collection as no standard referral pathway for obstetric care is present. Additionally, data acquisition was based on patient recall. Hence, some information is missing. However, the strength is that our center has a very good pool of women with Type-1 diabetes and data is recorded on specialized HMS at every visit.

## CONCLUSION

In this high-risk population, an increased frequency of unplanned pregnancies and cesarean sections along with inadequate pre-pregnancy care and suboptimal glycemic control, leads to compromised maternal and perinatal outcomes.

### Recommendation:

Pre-pregnancy counseling is essential in reproductive age group women with Type-1 diabetes. Optimal glucose control during conception and pregnancy mitigates adverse outcomes in Type-1 diabetes. A collaborative healthcare team, comprising of endocrinologists, obstetricians, dietitians, and diabetes educators, is crucial for optimizing outcomes for both mother and baby.

### Authors contribution:

**MR:** Concept and design, interpretation of data, edited and approved the final manuscript.

**SA:** Data collection, interpretation of data, manuscript writing, final editing.

**RN:** Data collection, manuscript writing, approved the final manuscript.

All authors are accountable for the integrity of work.
